# Retraction Note: Silibinin suppresses epithelial–mesenchymal transition in human non-small cell lung cancer cells by restraining RHBDD1

**DOI:** 10.1186/s11658-025-00728-4

**Published:** 2025-04-18

**Authors:** Suyan Xu, Hongyan Zhang, Aifeng Wang, Yongcheng Ma, Yuan Gan, Guofeng Li

**Affiliations:** Department of Pharmacy, Henan Provincial People Hospital, Department of Pharmacy of Central China Fuwai Hospital, Central China Fuwai Hospital of Zhengzhou University, Zhengzhou, 450003 Henan China

**Retraction Note:** **Cellular & Molecular Biology Letters (2020) 25:36 **10.1186/s11658-020-00229-6

The Editor-in-Chief has retracted this article because it contains data that overlaps with data from the following articles [1–4], the first of which was also published by *Cellular & Molecular Biology Letters*. In addition, an investigation conducted after the publication of these two articles, which were under consideration by the journal at the same time, found additional signs which raise concerns about the authorship of the two manuscripts and the circumstances of the research presented in them. The Editor-in-Chief therefore no longer has confidence in the results and conclusions presented in this article.

The authors have not replied to correspondence from the Publisher.
